# Effect of a Combined Stretching and Strength Training Program on Gait Function in Children with Cerebral Palsy, GMFCS Level I & II: A Randomized Controlled Trial

**DOI:** 10.3390/medicina55060250

**Published:** 2019-06-06

**Authors:** Merete Aarsland Fosdahl, Reidun Jahnsen, Kristin Kvalheim, Inger Holm

**Affiliations:** 1 Department of Clinical Neuroscience for Children, Division of Pediatric and Adolescent Medicine, Oslo University Hospital, 0424 Oslo, Norway; reijah@ous-hf.no (R.J.); kristin.kvalheim@helse-mr.no (K.K.); 2 Department of Interdisciplinary Health Sciences, Medical Faculty, University of Oslo, 0318 Oslo, Norway; inger.holm@medisin.uio.no; 3 Section of Research, Division of Orthopaedic Surgery, Oslo University Hospital, 0424 Oslo, Norway

**Keywords:** cerebral palsy, gait function, hamstrings stretching, progressive resistance training

## Abstract

*Background and objectives:* Ambulant children with cerebral palsy (CP) often develop impaired gait, and reduced active knee extension is often a part of the problem. This study aimed to evaluate the effect of a combined intervention program including stretching and progressive resistance exercise (PRE) targeting active knee extension on gait function, in children with spastic CP. *Materials and methods:* Thirty-seven children (21 boys, 16 girls, mean age 10.2 (±2.3) years), classified by Gross Motor Function Classification System I–III, were randomized to an intervention (*n* = 17) and a comparison group (*n* = 20). The intervention group received a 16-week combined exercise program (3 sessions per week) including stretching of hamstrings and PRE targeting the lower extremities, followed by a 16-week maintenance program (1 session per week). The comparison group received care as usual. Gait function was evaluated by three-dimensional gait analysis (3DGA); knee, hip and pelvic kinematics in the sagittal plane, step length and speed, Gait Deviation Index (GDI), and Six-Minute Walk test (6MWT) at 0, 16, and 32 weeks. *Results:* There were no statistically significant differences between the intervention group and the comparison group for any of the gait parameters measured at 16 and 32 weeks. There was a significant increase in gait distance measured by 6MWT within both groups; however, no differences between the groups were found. *Conclusion:* A 16-week combined stretching and PRE program followed by a 16-week maintenance program did not improve gait function in ambulant children with CP.

## 1. Introduction

Cerebral palsy (CP) is one of the most common causes of gait deviation in children. Children with CP start walking later than typically developed children and about 30% never walk independently [1,2]. This is caused by damage to the immature brain, which often results in primary impairments, like increased muscle tone, loss of selective motor control and impaired balance mechanisms, causing secondary impairments, such as muscle shortening, muscle weakness and decreased joint range of motion (ROM) [1,2]. These primary and secondary impairments often influence both the ambulation quality and capacity during childhood [1,3,4].

Studies using data based three-dimensional gait analysis (3DGA), including kinematics, kinetics, and spatial temporal gait parameters, show that gait function in children with CP deteriorates over time [3,5]. Gait is a complex activity and Gage et al. [1] describes the five prerequisites for normal gait as: stability in stands, foot clearance in swing, preposition of the foot in terminal swing, an adequate step length and energy conservation. To achieve all these five prerequisites, there has to be adequate muscle strength, joint position and segment alignment [1], and stretching and muscle strength training are assumed to be important for the maintenance and improvement of gait function [1,6].

Children with CP spend much of their childhood receiving physiotherapy focusing on optimal gait performance; hence choosing valid and effective treatment modalities is of great importance. Reduced muscle strength in CP is shown to be associated with impaired gait function and children with spastic CP, even the children who are mildly affected, have significantly lower limb muscle strength compared to typically developing (TD) children [1,7,8]. Studies have shown that muscle strength training, especially progressive resistance exercises (PRE) [9], improve muscle strength [10,11]; however, the increased strength does not seem to improve gait function [10,11,12]. Nevertheless, there are some studies indicating an effect on gait function after strength training [13,14,15,16]. In addition to muscle weakness, muscle spasticity and muscle shortening contribute to the restricted gait function [1,5]. Muscle contractures are shown to hamper long-term gross motor progress, while intensive training (≥3 times per week) enhances gross motor progress [17,18]. Muscle shortening is associated with joint stiffness and pain [19], and about one of four adolescents with CP experience knee pain [20]. Short hamstrings tend to cause restrictions on the knee extension at initial foot contact, and knee extension in mid-stance. As a bi-articular muscle it also tends to rotate the pelvis posteriorly [1]. Some muscles are more affected and hamstring shortening is shown to be more pronounced in children with lower functional levels [3,5,21], (classified by the Gross Motor Function Classification System (GMFCS) [22]) and with increasing age. 

McNee et al. [23] studied the lower limb extensor moment and underlined the importance of knee extension in mid-stands, because it seems to be essential for achieving an extending moment in the lower limb. The extensor moment in combination with the muscles stabilising the knee joint contributes to stability and smooth progression over the stationary foot, which is essential for the swing of the opposite leg and an optimal step length [1]. Mc Nee et al. [23] suggested that if there is an increased knee flexion, a disproportionate degree of support must be generated by the knee extensors. 

Stretching as the only physiotherapy treatment modality in CP is scarcely documented and the effect size is small [24,25,26]. However, muscle stretching is still commonly used [2], and it is mainly based on the assumption that stretching maintains or increases ROM [27]. To explore the effect of this practice, reviews conclude that the evidence is limited and more research with longer follow-up periods is needed [24,25,26].

Physiotherapy comprises different modalities, recognizing the fact that complex functional problems may need complex interventions [14]. Studies combining muscle strengthening and enhancing alignment and ROM have been asked for [14,23,28]. Thus, the aim of the present study was to evaluate if a 16-week combined hamstring stretching and PRE program, focusing on terminal knee extension and the extending muscles in the lower extremities, could improve kinematics and gait efficiency in children with spastic bilateral CP. A secondary aim was to evaluate if a 16-week maintenance program could preserve the possible gained improvements.

## 2. Materials and Methods

### 2.1. Study Design

**Figure 1:**
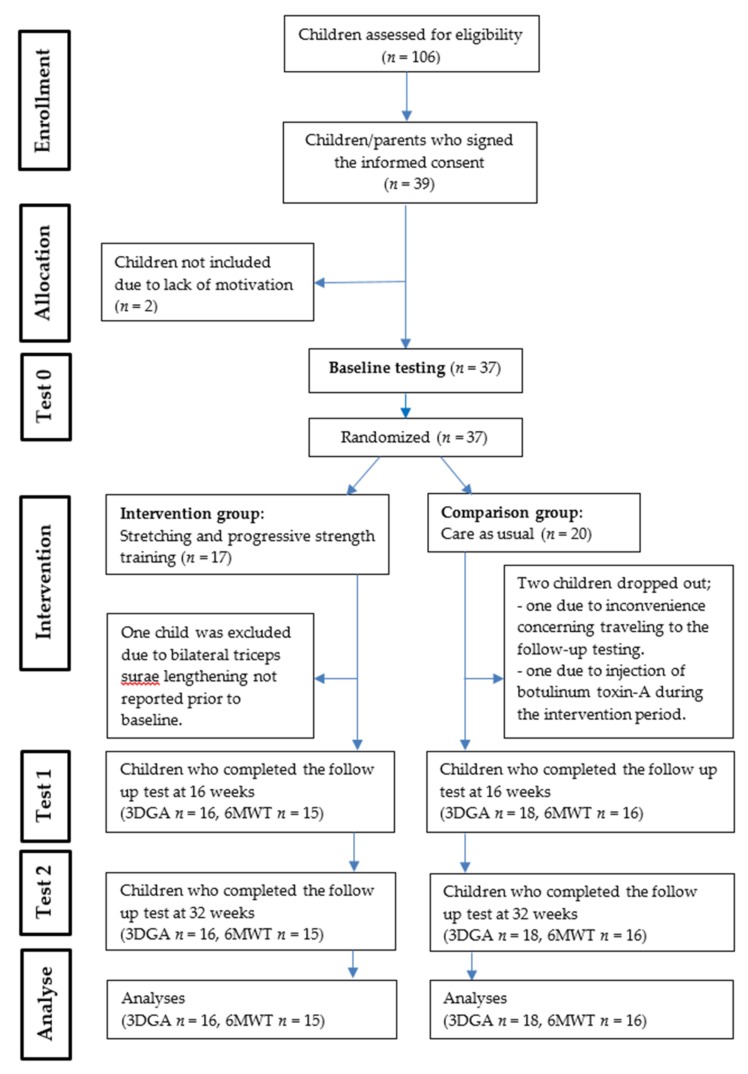
Flow-charge showing how the participants were moving through the study period. 3DGA: three-dimensional gait analysis, 6MWT: six-minute walk test.

The present study was a single-blind block randomized controlled trial. After baseline testing, the children were block randomized into two groups. The procedure was performed by an office administrator not engaged in the project, who opened sealed envelopes including blocks of four numbers. The participants were either allocated to an intervention group, performing a stretching and lower extremity PRE program (*n* = 17), or a comparison group receiving care as usual (*n* = 20) (Figure 1). The group allocation was assigned by the project manager who was not masked to the intervention. Assessments were completed at baseline (T0), after 16 (T1) and 32 (T2) weeks. The child’s local physiotherapist, who knew the child well, was responsible for the one-to-one intervention program. The assessors responsible for the testing were blinded to the randomization groups, and the children and their parents were told not to disclose their group affiliation. All the children and their parents gave their informed consent to participate in the study. The study was conducted in accordance with the Declaration of Helsinki, was approved on 26. November 2014 by the Regional Committee for Medical and Health Research Ethics, section South-East, the Commissioner for the Protection of Privacy in Research (2014/1766) and was registered in Clinical trial.gov (NCT02917330).

### 2.2. Participants

One hundred and six eligible children with spastic bilateral CP were identified by the CPOP, or by the patient register at the Motion Laboratory at Oslo University Hospital, and invited to participate in the study (Figure 1). 

The criteria for inclusion were (1) spastic bilateral CP, (2) age between 7 and 15 years, (3) GMFCS I–III, (4) able to walk 10 m indoors without walking aids, and (5) passive popliteal angle (PPA) ≥35° in the most affected leg. Exclusion criteria were (1) hamstring tenotomy, bilateral lengthening of the triceps surae, or any other surgical procedure in the lower limbs less than one year prior to inclusion. (2) Botulinum toxin-A-injections in the lower limbs the last six months prior to inclusion, (3) <0° dorsal flexion in the ankle joint, (4) <5° external rotation in the hips and (5) unable to cooperate or understand instructions.

### 2.3. Intervention

The children randomized to the intervention group followed a detailed program protocol including active and passive stretching of hamstrings [27] and PRE [9] focusing on the extending muscles in the lower extremities. The resistant training program was following the National Strength and Conditioning Association (NSCA) Guidelines [9] and modified by the recommendations from Verschuren et al. [29]. The stretching part of the program was a combination of passive and active muscle stretching exercises based on established techniques used in physiotherapy [27], the frequency chosen was based on previous published research studies [24,25,30] and clinical experience. The 16-week intervention program was performed three times per week: two sessions together with the physiotherapist and one home exercise session; 48 sessions in total. The children allocated to the comparison group received care as usual and their physiotherapists were by written information told not to introduce any new treatment modalities during the 32-week study period. 

To assure the quality and consistency of the stretching and PRE program, a detailed project protocol and an instructional film were distributed to the physiotherapists. They were also contacted and guided by a senior physiotherapist (project manager) not masked for the intervention, both before and during the intervention period.

The main intervention program (0–16 weeks) was performed 3 times per week. For a complementary description, see Fosdahl et al. [31]. Exercises were performed together with the physiotherapists, two times per week:

- Five-minute warm-up on a treadmill or a stationary bicycle.
- Physiotherapist assisted stretches:
  - Hamstring stretch was performed bilaterally with the child positioned supine, one leg flat on the bench and the contra lateral hip joint flexed to 90°. The physiotherapist supported the thigh and the child performed an active extension of the knee. Voluntary active knee extension was held for 5 s followed by the physiotherapist supporting and keeping the stretch, with constant stretching force as tolerated by the child at the end position for additional 40 s; 5 repetitions were performed.
  - If a short psoas was registered at baseline (≤5° extension) a psoas- stretch was performed with the child positioned prone. The child preformed an assisted active extension of one hip for 5 s, followed by the physiotherapist who supported and kept the hip extension at the end position for additional 40 s; 5 repetitions. There was 10–15 s rest between the stretches.
- Four PRE exercises:
  - Three multi-joint exercises with a loaded back-pack: (1) squats, (2) heel rise and (3) step-up on a stair.
  - One single-joint exercise was performed with the child positioned supine on a bench performed maximum knee-extension over a bolster. The physiotherapist applied manual resistance on the distal leg. It was therefore not possible to objectively control the load and progression applied.
  - The exercises were performed bilaterally with 2 min. breaks between the exercises

The three multi-joint exercises ((1), (2), (3)) were performed in an upright, axial loaded position wearing a back-pack, and focus on the terminal knee extension. To familiarize the children with the exercises, the back-pack was unloaded the first week, the second week the back-pack was loaded with weights or bottles filled with water. 

The amount of initial weight load followed the principles and recommendations suggested by Scholtes et al. (2008) [32], performing an eight-repetition maximum (8 RM) sit to stand exercise test, with a start load of about 35%, 30% and 25% of the child’s bodyweight, for GMFCS level I, II and III, respectively. The load was increased as the child became stronger, based on the 8 RM test [31] repeated every third week. The number of repetitions was progressively increased: 2 series of 12 repetitions (week 1 and 2), 3 series of 12 repetitions (week 3 to 5), 3 series of 10 repetitions (week 6 to 8) and after 8 weeks 3 series of 8 repetitions was performed. The weight load progression was administered and recorded by the local physiotherapist. A handrail was available for those who required any balance support. 

Exercises were performed at home, once a week:

- Passive hamstring stretch: sitting on a chair with one knee extended and the heel on the floor, leaning the trunk forward from the hip joint. The stretch was held for 45 s × 5, bilaterally.
- Strength exercise: squats (1) with a loaded back-pack. To familiarize with the two home exercises, the child was instructed by the physiotherapist the first three weeks. The following weeks the exercises were performed at home without guidance

The maintenance program (17–32 weeks) was performed once a week. The maintenance program was identical to the two above-described home exercises. In addition, both the intervention group and the comparison group received care as usual during the maintenance period. The numbers of physiotherapy, home exercises and care as usual sessions attended were registered by the physiotherapists and returned to the project manager by e-mail.

### 2.4. Outcome Measures 

The 3DGA variables were; sagittal plane kinematics from knee, hip and pelvic angle at foot strike, minimum knee flexion in stands, and the gait efficiency parameters: gait speed and step length. The Gait Deviation Index (GDI) [33] was calculated and in addition, the Six-Minute Walk Test (6MWT) [34,35] was performed. All tests, except for the 6MWT were administered by two senior therapists masked for the group allocation. The tests were performed in the Motion Laboratory at Oslo University Hospital and at Haukeland University Hospital in Bergen. Both test sites had a VICON motion laboratory (Vicon Motion Systems, Oxford, UK), with six 3D MX cameras, two 2D cameras and AMTI (Advanced Mechanical Technology, Inc., Watertown, MA, USA) force plates used for the 3DGA. Sixteen reflex markers were placed, on body landmarks according to the Helen Hays’ model [36] by the same assessor at every assessment. The children walked on a 10-meter walkway, at self-selected speed, until 5 trials of 3D-data with satisfactory quality were collected. These five trials were processed in Vicon Nexus 2.5 and gait events were processed in Vicon ProCalc 1.1. The second gait cycle from each of the five trials was selected, and mean values from the five cycles were calculated and used as the raw score from each gait event. 3DGA has an overall acceptable reliability with a measurement error in the sagittal plane between 2° and 4° [37], and a single assessor is shown to be more reliable then multiple assessors [38]. 

GDI is a gait index expressing the overall gait pathology derived from the 3DGA kinematic parameters into one single numeric measure. The values are ranged from 0–100, where 100 and above indicate absence of pathology. The GDI index was calculated using the GDI pipeline in Vicon Nexus 2.6. Mean GDI was calculated from the five trials collected.

Local physiotherapists, not involved in the treatment of the children, administered the 6MWT. They received a detailed description based on the American Thoracic Society’s guidelines [34]. The test was performed on a 15-meter walkway and standardized oral instruction was given. The 6MWT has been documented to have good test-retest [39] and between-tester reliability [40].

### 2.5. Statistical Analysis 

Data were analysed using the statistical analysis software program SPSS V25 for Windows (SPSS Inc, Chicago, IL, USA). Descriptive values are presented as means (± SD) (Table 1), and paired sample t-tests were used to calculate within group mean differences between baseline and 16 weeks, and between baseline and 32 weeks (Table 2). To compare baseline variables between the intervention and the comparison group, Student’s t-test was used for continuous, normally distributed data, and Chi square test for categorical variables. Two-tailed value of *p* < 0.05 was considered statistically significant. To evaluate mean differences between the two groups at 16 and 32 weeks, linear regression analysis with covariates correcting for baseline values (ANCOVA) was performed. Due to a wide age-span, age was added as a covariate in the model. Seven random missing values from the 6MWT were substituted using single imputation by last value carry forward [41].

The outcome variables reported in the present paper are secondary variables derived from a recent RCT [31], and the sample size calculation was based on the primary outcome variable, the passive popliteal angle. In order to achieve 80% test power, at least 16 participants in each group had to attend the first follow up test after 16 weeks. 

## 3. Results

According to the randomization procedure 17 children were allocated to the stretching and PRE program and 20 children to the comparison group. Three children were excluded before the first follow-up test (Figure 1). Thirty-four children completed the 3DGA at all the three test sessions (T0, T1, T2). However, due to practical reasons only 31 children performed the 6MWT at all test points. There were no significant differences between the two groups at T0 for any of the baseline variables (Table 2).

The compliance registration form was answered by 81% of the physiotherapists. For the intervention group, the compliance rates were 79% (25 sessions ±4), 76% (12 sessions ±5) and 73% (35 sessions ±6) for the sessions together with the physiotherapist, the home exercise sessions, and total number of sessions, respectively. The compliance rate for the maintenance program was 72% (13 sessions ±4). The most frequent reasons for absence from training were illness, vacations and conflicting appointments. At baseline, two children in the intervention group showed a short psoas muscle, (≥5° extension) and for that reason psoas stretches were included as a part of the intervention. During the study period, 60% of the children in the comparison group received physiotherapy as usual, ranging from one to two sessions per week. There was high variation in the physiotherapy modalities given. Five children received strength and/or stretching exercises, and ten children received functional training, swimming or horseback riding. Descriptive values and differences within groups for all outcome measures at T0, T1 and T2 are presented in Table 2. For the 6MWT, there were significant changes within both groups both at T1 and T2. There were no significant within group changes for any of the other gait variables (Table 2).

For the kinematic gait variables, gait speed, step length, GDI and 6MWT, there were no significant mean differences between the intervention group and the comparison group, neither at T1 nor at T2 (Table 3).

## 4. Discussion

The results from the present study showed that a 16-week hamstring stretching and PRE program and a 16-week maintenance program did not result in any significant mean difference between the intervention group and the comparison group for any of the gait parameters measured (Table 3). To our knowledge, no previous published study has evaluated the effect of a combined hamstring stretching and PRE program, targeting the extending muscles in the lower extremities, on different gait parameters. The rationale for introducing this combined intervention program was the assumption that improvement and maintenance of knee extension and muscle strength in the lower extremities is essential to optimize the prerequisites of gait in children with CP [1]. Adequate active terminal knee extension is important for maintaining strength, stability and dynamic control both in mid stance and terminal swing face. Previous studies, including children with CP with short hamstrings and crouch gait, have concluded that there is a need for interventions where the goal is to both preserve muscle strength and maintain ROM [14,28].

Active terminal knee extension is often reduced in children with CP [1,7]. Onpuu et al. [42] compared typically developing children with children with CP, and found that in the typically developing group the maximum knee angle at foot strike was 10° flexion, and 90% of the ambulatory children with CP (GMFCS I-III) showed more than 10° knee flexion at foot strike [42]. This is in line with the baseline values in the present study, showing a mean knee flexion angle at foot strike of 16° (±6) in both groups. Thompson et al. [7] studied isometric muscle power in 50 children with spastic CP, GMFCS levels I-III, and found that at 30° knee flexion, the knee extensors were the relatively weakest muscle group compared to typically developing children, worsening with decreasing gait function. Cloodt et al. [43] found that a knee joint contracture was associated with short hamstrings and therefore argued that maintaining hamstring length is important for reducing the risk of knee contractures. 

The exercise program included in the present study primarily focused on increasing and maintaining the hamstring flexibility and strengthening of the muscles responsible for the active terminal knee extension. Despite this specific focus, there were no improvements in knee kinematics, either at foot strike, at mid stance, or in the GDI (Table 3) for the intervention group at T1, and there was even a small decrease at T2. In the comparison group, there were slight improvements in the hip and knee kinematics at T1 and T2 (Table 2). These changes may be explained by a relatively small number of children included in the study, and normal variation (measurement errors) in the 3D-measurements between the different time points (McGinley 2009 [37]). We are aware that the present study is in line with previous published studies showing minor or no effect and even negatively influence on the kinematic variables following muscle strength training [8,10,14]. However, these studies included shorter intervention periods, and it was discussed if programs with longer lasting intervention periods, coupled with other interventions and monitoring of the hamstring muscle length [14] might have shown a more positive result. Our pre-study hypothesis was that by prolonging the intervention period up to 16 weeks, and adding stretching of the hamstring muscles, there might be a better rationale for improving the knee kinematics, thereby resulting in improved gait efficiency measured by increased step length and gait speed. Despite this prolonged intervention period, no significant improvements in gait function were found (Table 3). The chosen exercises included in the present intervention program followed the recommendations for youth resistance training published by NSCA [9] with modifications recommended for children with CP [29], and established physiotherapy methods for manual stretching were used. The intensity of the static stretch was instructed to be a 40 second continuous end-point stretch not painful to the child. However, the amount of load applied by the physiotherapist was not possible to quantify. 

Dose and intensity according to the NSCA guidelines were described in the exercise protocol. The progression of weight load in the back-packs was adjusted by the 8 RM test performed by the physiotherapist every third week. The physiotherapists were guided by a project manager; however, the 16 different physiotherapists may have different understanding of and experience with the NSCA guidelines, PRE training and how to guide and motivate the child, resulting in variations in both dose and intensity applied. In the single-joint knee extension exercises, the weight load on the distal leg was controlled by the physiotherapist and through feedback from the child and the hands-on felt muscle response. Hence, the quality of this exercise depended on the physiotherapist’s individual hands-on skills. 

The individual experience and knowledge of the physiotherapists may to some extent, have interfered with the effectiveness of the exercises. A pre-study course for the physiotherapists in the intervention group might have increased the quality and consistence of the intervention; however, due to geography and lack of time it was difficult to arrange. Another reason for the insignificant group differences in the present study may be lack of gait specific exercises. In a recent intervention study by van Vulpen et al. [16], children with CP (GMFCS levels I and II) performed functional high velocity resistance training, with progressive external resistance, to improve muscle strength and walking capacity. The study showed significant effect on walking capacity, muscle strength and increased passive ROM in the ankle joints. The results indicate that functional strength exercises performed with higher velocity might be more suitable for improving gait function.

The exercises included in the present exercise program emphasized active knee extension both in a standing position and unloaded, lying supine on a bench. To achieve a carryover-effect from impairment-focused strength training to improve active knee extension and stability in the gait cycle, specific gait training should probably have been a part of the intervention program. In a systematic review and meta-analysis by Moreau at al. [12], they concluded that gait training was more effective than strength training in improving gait speed. However, they did not evaluate any impact on kinematic variables.

In a recent study, Fitzgerald et al. [35] studied 145 children with CP and presented 6MWT reference values for the different GMFCS levels with a mean of 377 m. The results from the present study correspond well with Fitzgerald’s findings (Table 2). There was no significant difference between the intervention and the comparison groups at T1 or T2 (Table 3). However, there were significant changes within both groups at T1 and the change remained unchanged at T2 for both groups (Table 2). Nevertheless, the changes were not above the minimal detectable change, as documented by Thompson et al. [44]. The reliability of the 6MWT for children with CP is documented [39,44], and Maher et al. [39] stated that a practice test before the first test is not necessary. However, the significant changes in both groups registered in the present study might have been influenced by a learning effect or the children might have been more motivated when they were familiar with the test. In addition, 60% of the children in the comparison group also received physiotherapy (care as usual), which might also have influenced the results. The 6MWT was the only test performed locally, and the same child was tested by the same physiotherapist on all three occasions. Even though the test situation followed the international guidelines described by the American Thoracic Society [34], and the inter-rater reliability is shown to be acceptable [40], there is a risk that the test situations differed to some degree, but there should not be any reason for a biased group difference. 

There are some limitations to the present study. First, only one child classified at GMFCS level III was recruited, indicating that the results are not applicable to children at GMFCS level III. Second, for an optimal and more consistent guiding and implementation of the intervention, there should have been one physiotherapist responsible for all the children in the intervention group. The geographical distribution of the children made that impossible to implement. Third, to limit the number of tests the child had to perform on the day visiting the hospital, the 6MWT was administered locally by a different assessor for each child. However, for some children and local physiotherapists, the testing became difficult to carry out, resulting in some missing tests, making the 6MWT results incomplete. A fourth limitation was the lack of a more detailed mapping of the content of the intervention given to the children in the comparison group. The physiotherapy modalities registered revealed variations in content and frequency, and some of the modalities used may have interfered with the results. To cope with this, a comparison group not receiving any kind of physiotherapy during the intervention period could have been included; however, this was considered unethical.

## 5. Conclusions

The results from the present study showed that a 16-week combined hamstring stretching and PRE program, followed by a 16-week maintenance program did not result in any difference in change between the intervention and the comparison group in any of the gait parameters evaluated. However, the 6MWT showed significant improvements within both groups after 16 weeks. Future studies aiming at improving specific impairments in gait function should probably include some kind of gait specific exercises. 

## Figures and Tables

**Table 1:** Descriptive characteristics of the participants at baseline.

Variables	Intervention Group (*n* = 17)	Comparison Group (*n* = 20)
Gender (boys/girls)	7/10	14/6
Age (years)	10.4 ± 2.3	10.0 ± 2.3
Height (cm)	141.3 ± 16.5	141.4 ± 12.8
Body weight (kg)	37.6 ± 13.3	39.9 ± 12.9
BMI (kg/m^2^)	17.8 ± 3.6	19.7 ± 4.7
GMFCS I/II/III	10/7/0	12/7/1

Values presented as mean ± SD; *n*: number of participants; BMI: Body Mass Index; GMFCS: Gross Motor Function Classification System.

**Table 2:** Gait parameters at baseline (T0), 16 weeks (T1) and 32 weeks (T2).

Gait Parameters	Intervention Group					
	*n*	T0	T1	T0–T1	T2	T0–T2
		Mean ± SD	Mean ± SD	Mean Diff ± SD	Mean ± SD	Mean Diff ± SD
Knee angle, foot strike (deg)	16	16.1 ± 6.8	16.1 ± 8.5	−0.01 ± 4.4	17.5 ± 10.5	−1.4 ± 6.1
Hip angle, foot strike (deg)	16	41.1 ± 12.1	42.6 ± 11.7	−1.5 ± 4.7	41.7 ± 10.2	−0.6 ± 5.6
Pelvic angle, foot strike (deg)	16	16.8 ± 7.3	16.9 ± 7.6	0.5 ± 3.1	15.6 ± 7.8	0.3 ± 3.1
Min knee angle, stands (deg)	16	5.7 ± 8.7	6.0 ± 8.9	−0.3 ± 4.8	6.4 ± 9.8	−0.7 ± 6.1
Step length (cm)	16	52.7 ± 8.2	54.0 ± 9.8	−1.3 ± 5.5	55.8 ± 10.6	−3.2 ± 5.1
Speed (m/s)	16	1.05 ± 0.2	1.1 ± 0.2	−0.05 ± 0.1	1.04 ± 0.3	0.18 ± 0.3
Six-Minute Walk Test (m)	15	390.5 ± 106.9	436.2 ± 114.8	−45.7 ± 55.4 *	441.6 ± 121.6	−51.1 ± 72.8 *
Gait deviation index	16	78.8 ± 11.1	79.2 ± 11.2	−0.4 ± 4.4	79.5 ± 11.7	−0.7 ± 6.0
	**Comparison Group**					
	***n***	**T0**	**T1**	**T0**–**T1**	**T2**	**T0**–**T2**
		**Mean ± SD**	**Mean ± SD**	**Mean Diff ± SD**	**Mean ± SD**	**Mean Diff ± SD**
Knee angle, foot strike (deg)	18	17.5 ± 9.0	15. 0 ± 10.6	2.6 ± 5.2	14.8 ± 10.9	2.6 ± 6.4
Hip angle, foot strike (deg)	18	39.9 ± 7.5	37.8 ± 7.7	2.1 ± 7.2	38.0 ± 8.6	1.9 ± 6.5
Pelvic angle, foot strike (deg)	18	14.3 ± 5.3	13.2 ± 3.9	1.1 ± 3.9	14.2 ± 3.9	0.1 ± 4.1
Min knee angle, stands (deg)	18	5.9 ± 9.3	4.0 ± 10.7	1.8 ± 5.0	3.4 ± 11.5	2.4 ± 5.2
Step length (cm)	18	51.6 ± 9.5	51.2 ± 9.8	0.4 ± 5.4	51.3 ± 10.4	0.3 ±7.4
Speed (m/s)	18	1.02 ± 0.2	0.97 ± 0.3	0.05 ± 0.2	1.03 ± 0.2	−0.01 ± 0.2
Six-Minute Walk Test (m)	16	349.9 ± 112.7	405.2 ± 123.5	−55.4 ± 55.5 *	406.5 ± 133.9	−56.6 ± 59.6 *
Gait deviation index	18	80.0 ±9.7	79.1 ± 15.2	0.8 ± 7.14	79.0 ± 11.6	1.01 ±5.9

Group raw values presented as mean ± SD at baseline (T0), 16 weeks (T1) and 32 weeks follow-up (T2), and mean within group difference ± SD between T0–T1 and T0–T2 (pared sample t-test), Statistically significant *: *p* < 0.05; *n*: number of participants; deg: degrees; flex: flexion; max: maximum; min: minimum.

**Table 3:** Comparison of mean difference between the intervention and comparison group at T1 and T2 when adjusted for baseline and age, in the linear regression model (ANCOVA).

Gait Parameters	T0–T1				T0–T2	
	*n*	Mean Difference (95% CI)	*p* Value	*n*	Mean Difference (95% CI)	*p* Value
Knee angle, foot strike (deg) Hip angle, foot strike (deg) Pelvic angle foot strike (deg) Minimum knee angle in stands (deg) Step length (cm) Speed (m/s) Six-Minute Walk Test (m) Gait deviation index	34 34 34 34 34 34 31 34	−2.4 (−5.8 to 1.0) −3.7 (−7.9 to 0.5) −1.6 (−3.9 to 0.7) −2.2 (−5.7 to1.4) −1.5 (−5.3 to 2.2) −0.1 (−0.2 to 0.05) 10.6 (−29.3 to 50.6) −1.0 (−5.3 to3.3)	0.161 0.081 0.172 0.228 0.408 0.188 0.590 0.650	34 34 34 34 34 34 31 34	−3.9 (−8.5 to 0.6) −2.8 (−6.9 to 1.3) 0.6 (−1.8 to 2.9) −3.2 (−7.3 to 1.0) −3.3 (−7.9 to 1.2) −0.02 (−0.02 to 0.2) 7.2 (−43.3 to 57.7) −1.4 (−5.6 to 2.8)	0.088 0.175 0.638 0.128 0.149 0.778 0.772 0.504

T1: 16 weeks follow-up, T2: 32 weeks follow-up, n: number of participants, CI: confidence interval, deg: degrees, statistically significant: *p* < 0.05.
